# The Aging Process: A Metabolomics Perspective

**DOI:** 10.3390/molecules27248656

**Published:** 2022-12-07

**Authors:** Alex Castro, Étore F. Signini, Juliana Magalhães De Oliveira, Maria Carolina Bezerra Di Medeiros Leal, Patrícia Rehder-Santos, Juliana C. Millan-Mattos, Vinicius Minatel, Camila B. F. Pantoni, Regina V. Oliveira, Aparecida M. Catai, Antônio G. Ferreira

**Affiliations:** 1 Department of Chemistry, Universidade Federal de São Carlos, São Carlos 13565-905, Brazil; 2 Department of Physiotherapy, Universidade Federal de São Carlos, São Carlos 13565-905, Brazil

**Keywords:** metabolism, metabolome, nuclear magnetic resonance, liquid chromatography-high-resolution mass spectrometry

## Abstract

Aging process is characterized by a progressive decline of several organic, physiological, and metabolic functions whose precise mechanism remains unclear. Metabolomics allows the identification of several metabolites and may contribute to clarifying the aging-regulated metabolic pathways. We aimed to investigate aging-related serum metabolic changes using a metabolomics approach. Fasting blood serum samples from 138 apparently healthy individuals (20–70 years old, 56% men) were analyzed by Proton Nuclear Magnetic Resonance spectroscopy (1H NMR) and Liquid Chromatography-High-Resolution Mass Spectrometry (LC-HRMS), and for clinical markers. Associations of the metabolic profile with age were explored via Correlations (r); Metabolite Set Enrichment Analysis; Multiple Linear Regression; and Aging Metabolism Breakpoint. The age increase was positively correlated (0.212 ≤ r ≤ 0.370, *p* < 0.05) with the clinical markers (total cholesterol, HDL, LDL, VLDL, triacylglyceride, and glucose levels); negatively correlated (−0.285 ≤ r ≤ −0.214, *p* < 0.05) with tryptophan, 3-hydroxyisobutyrate, asparagine, isoleucine, leucine, and valine levels, but positively (0.237 ≤ r ≤ 0.269, *p* < 0.05) with aspartate and ornithine levels. These metabolites resulted in three enriched pathways: valine, leucine, and isoleucine degradation, urea cycle, and ammonia recycling. Additionally, serum metabolic levels of 3-hydroxyisobutyrate, isoleucine, aspartate, and ornithine explained 27.3% of the age variation, with the aging metabolism breakpoint occurring after the third decade of life. These results indicate that the aging process is potentially associated with reduced serum branched-chain amino acid levels (especially after the third decade of life) and progressively increased levels of serum metabolites indicative of the urea cycle.

## 1. Introduction

Aging is a natural biological phenomenon, characterized by a gradual and progressive decline of physiological and metabolic functions at multiple levels (molecular, organellar, cellular, tissue, and organic), which is influenced by genetic, environmental, and lifestyle factors, leading to impairment of the general functions of the organism, and increased vulnerability to death [1,2,3].

Metabolically, the aging process involves extensive alterations in body composition and insulin resistance, as well as promotes physiological declines in multiple signaling pathways including growth hormone, insulin/insulin-like growth factor 1 (IGF-1), and sex steroids regulation [4,5]. Aging also affects several biochemical processes, such as inflammation [6], proteostasis [7], oxidative stress response [8], excretion [9], and energy metabolism [10].

Studies show that aging is the main risk factor associated with several morbidities such as cardiovascular disease, diabetes, neurodegenerative conditions, cancers, and other prevalent malignancies [11,12]. In this sense, understanding the metabolic and biochemical context of aging can contribute to the discovery of new biomarkers and the development of predictive models capable of helping clinicians to recognize people at high risk of developing diseases or with poor health conditions [4,13].

In this perspective, metabolomics has emerged as a powerful tool for characterizing phenotypes, identifying metabolites, metabolic pathways, and new biomarkers related to aging [1,3,13,14,15]. Metabolomics allow the characterization and quantification of a wide range of compounds and metabolites in different biological systems, under specific time and conditions, typically using techniques such as proton nuclear magnetic resonance spectroscopy (1H NMR) and liquid chromatography coupled to high-resolution mass spectrometry (LC-HRMS) [16,17,18]. Metabolites are intermediate cellular products of metabolic reactions, which reflect the final response to genomic, transcriptomic, proteomic, or environmental changes in a biological system [19,20].

Recent studies have shown that metabolite profiles may reflect health and biological aging in humans [21,22,23]. Lower levels of carnitines and citrate cycle intermediates have been associated with higher biological age, indicating a reduced mitochondrial performance with aging evidenced by a decreased capacity for fatty acid utilization and adenosine triphosphate (ATP) production [22]. Additionally, people living in a more longevous region, characterized by a high centenarian incidence, tend to exhibit healthier aging metabolic patterns compared to those from a less longevous region, evidenced by elevated plasma levels of citrate, tyrosine, choline, carnitine, valine, as well as lower contents of very-low-density lipoprotein (VLDL), lactate, alanine, N-acetyl glycoprotein, trimethylamine oxide, α-glucose, and β-glucose [23]. From a systemic metabolic perspective, the key characteristics for longevity have been associated with the proper regulation of amino acid, lipid, carbohydrate, citrate cycle, and redox metabolisms [3,22,23,24].

Despite substantial research activities in recent decades, the precise biological mechanisms related to aging and longevity are still not fully understood [2,4,9]. Many metabolomic studies have treated age as a covariate [25,26], investigated metabolomic age scores related to biological or chronological age [13,22,27], and compared groups of different ages, even including centenarian people [23,24]. Although current studies have contributed to the advancement in the discovery of aging-regulated metabolic pathways, for a broad coverage of the metabolome and their metabolic networks, it is important to consider the use of complementary analytical platforms (e.g.,: 1H NMR or LC-HRMS), which is a limitation in most previous studies on this topic [9]. Furthermore, to our knowledge, no studies have shown when the greatest disturbance in metabolism is expected to occur with aging. 

Therefore, the aim of this study was to identify which metabolites in human serum are associated with aging, highlighting the main regulated metabolic pathways, as well as identifying a breakpoint in metabolism with aging using a metabolomics approach based on 1H NMR and LC-HRMS. Identifying the moment of the most significant disturbance in metabolism with aging has implications for the adoption of early medical interventions and the development of strategies for personalized treatment aimed at healthy aging.

## 2. Results

### 2.1. Metabolomics Data

The 1H NMR and LC-HRMS techniques yielded 47 and 128 serum compounds, respectively, which were included in the final data analysis. The significant compounds [(*p* < 0.05 and <False Discovery Rate (FDR)] related to the aging process were selected and their identification presented in detail (Tables S1 and S2). The selected compounds determined by 1H NMR were 3-hydroxyisobutyrate, asparagine, isoleucine, leucine, valine, aspartate, and ornithine, while those determined by LC-HRMS were tryptophan and other three unknown compounds.

### 2.2. Participant Characteristics

The total study sample consisted of 138 apparently healthy (without any health conditions, such as cardiovascular, respiratory, musculoskeletal, metabolic, and neurological issues) and untrained individuals, randomly split into: Training sample (*n* = 105), 58 men (55.2%) and 47 women (44.8%) with a median age of 42 years old (range: 20 to 69 years); and a Test sample (*n* = 33, validation study subgroup), 19 men (57.6%) and 14 women (42.4%) with a median age of 40 years old (range: 22 to 70 years). There were no significant differences between the Training and Test samples for physical and clinical characteristics of participants, and male-to-female ratio (*p* > 0.05 for all, Table 1).

### 2.3. Association of Physical and Clinical Characteristics of the Participants, and Metabolomic Profile with Age

**Figure 1:**
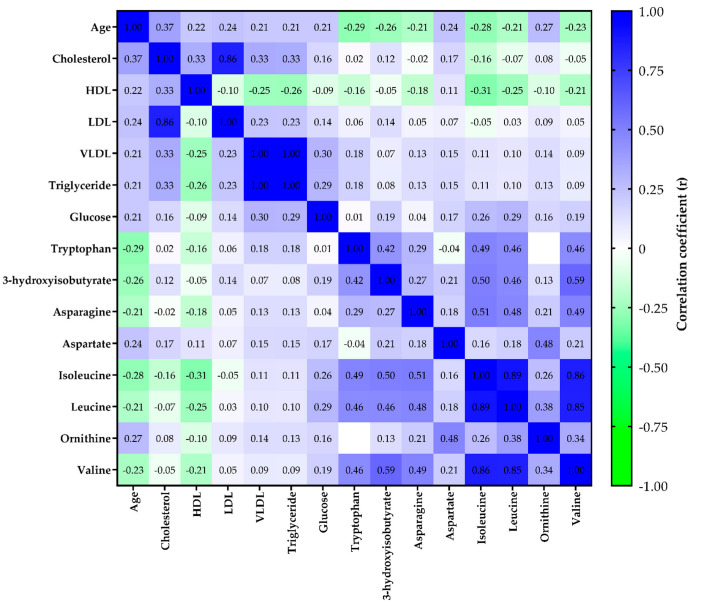
Overall correlation heatmap between age, clinical markers, and metabolite levels. Blue and green colors represent positive and negative correlations (r), respectively. High-density lipoprotein (HDL); Low-density lipoprotein (LDL); Very low-density lipoprotein (VLDL).

Body mass index (BMI) was positively correlated with age increase (r = 0.225, *p* = 0.021), but not with sex (*p* = 0.408). Therefore, subsequent correlation analyses were adjusted only for BMI. The age increase was positively correlated with total cholesterol (r = 0.370, *p* < 0.001), high-density lipoprotein (HDL, r = 0.219, *p* = 0.034), low-density lipoprotein (LDL, r = 0.243, *p* = 0.018), VLDL (r = 0.214, *p* = 0.039), triacylglyceride (r = 0.212, *p* = 0.040), and glucose (r = 0.213, *p* = 0.039) levels (Table S3, Supplementary Materials). For metabolomics, the age increase was negatively correlated with tryptophan (r = −0.285, *p* = 0.005), 3-hydroxyisobutyrate (r = −0.257, *p* = 0.013), asparagine (r = −0.214, *p* = 0.038), isoleucine (r = −0.280, *p* = 0.006), leucine (r = −0.215, *p* = 0.038), and valine (r = −0.225, *p* = 0.029), but positively, with aspartate (r = 0.237, *p* = 0.021) and ornithine (r = 0.269, *p* = 0.009) (Tables S4 and S5). All *p*-values reported herein were False Discovery Rate (FDR)-corrected for multiple hypothesis testing. Therefore, variables with *p*-values < 0.05 and < FDR-corrected *p*-values were considered statistically significant and retained for further analysis. The overall correlation heatmap between age, clinical markers, and metabolite levels is presented in Figure 1. The metabolites significantly associated with age, obtained by 1H NMR and LC-HRMS were also graphically presented in Figure S1.

### 2.4. Metabolite Set Enrichment Analysis

**Figure 2:**
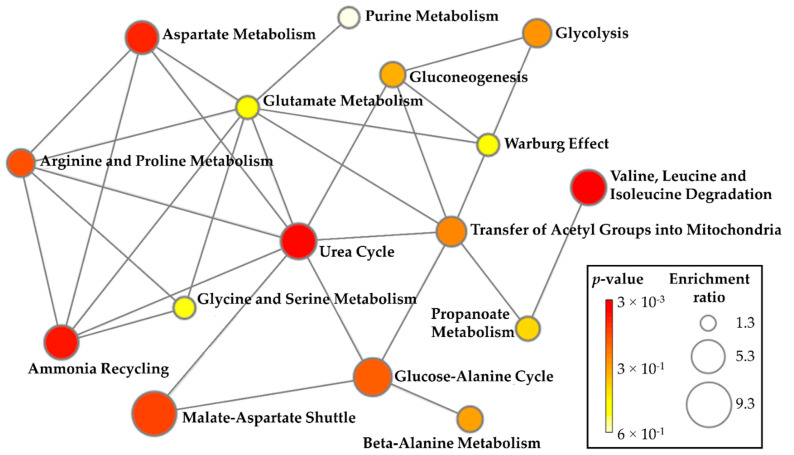
Metabolite Set Enrichment Analysis. The size and color (varying from red to white) of each circle represent the pathway enrichment ratio (computed by Hits/Expected hits) and *p*-value, respectively. The most enriched pathways enjoying connections were labeled.

For pathway enrichment analysis, lipids and metabolites that significantly correlated with age were used (triacylglyceride, cholesterol, glucose, 3-hydroxyisobutyrate, asparagine, aspartate, isoleucine, leucine, l-tryptophan, ornithine, and valine). A total of three distinct pathways were identified and significantly related to aging: valine, leucine, and isoleucine degradation (hits: valine, leucine, isoleucine, and 3-hydroxyisobutyrate); urea cycle (hits: aspartate and ornithine); and ammonia recycling (asparagine and aspartate). The complete list of identified pathways is summarized in detail in Table S6. The metabolic pathways and their connections with the most enriched pathways related to the aging process are labeled in Figure 2.

### 2.5. Summary of Key Metabolites Associated with Aging

Metabolites significantly selected in the previous two steps (correlation and pathway enrichment analysis) were further analyzed by a multiple linear regression model adjusted by BMI to determine the overall contribution of the serum metabolic levels on the aging process.

The multiple linear regression model demonstrated that 3-hydroxyisobutyrate (β = −3.3, *p* = 0.032), isoleucine (β = −3.5, *p* = 0.018), aspartate (β = 3.1, *p* = 0.028), and ornithine (β = 3.0, *p* = 0.041) levels explained 27.3% adjusted for BMI (r_multiple_ = 0.506). To further assess the replicability and stability of our findings, we performed a similar analysis in the Test sample achieving similar results (r_multiple_ = 0.605).

### 2.6. Identifying the Breakpoint in Metabolism Related to Aging

**Figure 3:**
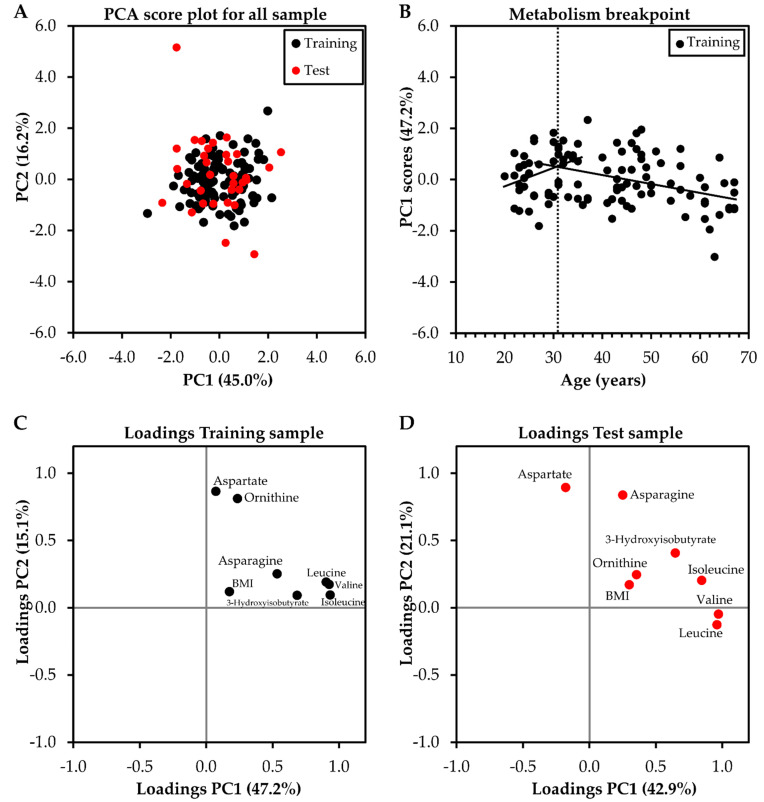
Score (**A**) and loading (**C**,**D**) plots of Principal Component Analysis (PCA) of main serum metabolites related to aging. Illustration of the metabolism breakpoint (**B**) from the intersection (dashed line) between the two straight lines (age vs. PC1 scores). Black and red circles represent Training and Test samples, respectively.

Metabolites selected from steps 1 and 2 (3-hydroxyisobutyrate, asparagine, aspartate, isoleucine, leucine, ornithine, and valine) with addition of BMI were also analyzed by a principal component analysis (PCA) to extract a representative score of the main factors of variability in the metabolism weighted for each metabolite and BMI. The higher variability in the metabolism was explained by PC1 (Figure 3A) with higher factor loadings observed for valine (Training sample = 0.926; Teste sample = 0.960), leucine (Training sample = 0.904; Teste sample = 0.971), isoleucine (Training sample = 0.933; Teste sample = 0.845), and 3-hydroxyisobutyrate (Training sample = 0.684; Teste sample = 0.646) (Figure 3C,D). Both Training and Test samples presented a similar grouping of variables with more significant loadings for valine, leucine, isoleucine, and 3-hydroxyisobutyrate in PC1, demonstrating the replicability of the main results.

Interestingly, there was a breakpoint in the linear relationship between age and PC1 scores, as observed by the intersection between the two straight lines around 32 years (Figure 3B).

## 3. Discussion

This study showed that age increase was positively associated with the clinical markers (total cholesterol, HDL, LDL, VLDL, triacylglyceride, and glucose levels); negatively associated with tryptophan, 3-hydroxyisobutyrate, asparagine, isoleucine, leucine, and valine levels, but positively with aspartate and ornithine levels. These metabolites resulted in three enriched pathways including valine, leucine, and isoleucine degradation, urea cycle, and ammonia recycling. Additionally, serum metabolic levels of 3-hydroxyisobutyrate, isoleucine, aspartate, and ornithine explained 27.3% of the age variation adjusted by BMI, with the aging metabolism breakpoint occurring after the third decade of life with branched-chain amino acids being the main source of variation in the metabolome.

For clinical markers, the positive association of the total cholesterol, HDL, LDL, VLDL, triacylglyceride, and glucose levels with age have been extensively demonstrated in the literature, mainly from twenty to sixty years old for most of them [28,29], which corroborates the age range considered in our study. These results demonstrate the importance of continuous monitoring of these clinical markers with aging, since very high total cholesterol, LDL, triacylglyceride, and glucose levels are associated with additional risks of cardiovascular disease, coronary heart disease, or even all-cause mortality [30,31,32,33].

Interestingly, several amino acids and 3-hydroxyisobutyrate were negatively associated with aging. The serum levels of the branched-chain amino acids (BCAAs: leucine, isoleucine, and valine) are expected to decrease in heathy individuals due to the involuntary loss of muscle mass with aging [34,35], a phenomenon called sarcopenia [36]. The BCAAs are the most abundant amino acids in proteins and are involved in the maintenance of skeletal muscle [35,37]. This argument can be supported in part by the positive association observed between BMI and aging. Although BMI does not directly reflect the distribution of lean body mass or muscle mass [38], the increase in BMI with aging in healthy and untrained individuals, as in the present study, is expected to occur mainly due to the gain in body fat mass and reduction in muscle body mass [38,39,40]. An intermediate in valine degradation, 3-hydroxyisobutyrate regulates the trans-endothelial flux of fatty acids; its lower level with aging is also in accordance with lower levels of valine [41]. The excessive catabolic flux of BCAAs can lead to a range of adverse cardiometabolic risk factors with aging, including obesity, insulin resistance, and dyslipidemia [41,42]. Curiously, the breakpoint in the metabolism occurred after the third decade of life, with BCAAs being the main ones responsible for modifications. This finding is supported by substantial reduction in the production of IGF-1, the main mediator for the trophic effects of growth hormone, that plays an important role in maintaining lean mass and bone mass [43], accompanied by a decline in the relative fat free mass, especially after the third decade of life [44,45]. 

Tryptophan and asparagine also were negatively associated with aging in agreement with other studies [37,46,47]. Tryptophan is a glucogenic and ketogenic essential amino acid which is a precursor for the neurotransmitter serotonin and melatonin that regulates circadian rhythms [37], and a precursor for the nicotinamide adenine dinucleotide (NAD+) whose decrease with aging is associated with metabolic and neurodegenerative diseases and various cancers [48]. The decrease in serum tryptophan with aging and the increase in its toxic catabolites have been attributed likely due to increased levels of the enzyme indoleamine-2,3-dioxygenase (IDO). IDO breaks down tryptophan to kynurenine in tissues outside the liver induced by pro-inflammatory cytokines and superoxide which increase with aging [37,49]. Asparagine is a glutamine-derived metabolite. Although the cause of its decrease in blood serum with aging is not well understood, asparagine plays an important role in blood vessel formation [50], proliferating cells, regulation of protein and nucleotide synthesis [51], and coordinates cellular homeostatic responses with metabolic fuel reserves and availability [50].

On the other hand, aspartate and ornithine were positively associated with aging as also observed in previous studies [9,46,52]. Both these amino acids play an important role in the urea cycle. While aspartate is an important carrier of nitrogen atoms for the urea cycle, ornithine is an intermediate in this cycle [53]. An possible interpretation of our finding is that the mechanisms of excretion of these metabolites through the urea cycle may be more compromised with increasing age due to the decrease in the rate of clearance of urea by the kidney [54], but not on a pathological level since our participants presented clinical profiles indicative of normal functioning kidneys (based on serum urea and creatinine levels). This reasoning can be supported since a greater efficiency of this cycle is expected to reflect in the higher flux of urea excretion, resulting in lower levels of these amino acids [15], contrary to what was observed in the present study with aging. Interestingly, we only observed a trend (*p* = 0.064, Table S3) toward an increase in serum urea levels with aging, however, previous studies have confirmed this positive association [34,55]. There is evidence that the accumulation of intermediate metabolites from the urea cycle is toxic for hepatocyte mitochondria due to the toxicity of ammonia [56]. 

In summary, our findings demonstrate that aging-induced changes in metabolism are related to the increased regulation of the valine, leucine, and isoleucine degradation pathway, especially after the third decade of life, which is accompanied by a progressive decrease in the ability of the urea cycle in excreting its degradation by-products. These results contribute to determining the metabolic underpinning of aging and have potential clinical implications for health monitoring as they mark important changes in metabolism, which may be useful for carrying out early therapeutic, pharmacological, and dietary interventions, aiming to mitigate possible deleterious effects of aging, such as loss of muscle mass and reduced liver/kidney efficiency.

Some limitations and strengths of the present study should be highlighted. Although, metabolic sex differences have been extensively documented in the literature [46,47,57,58], it was not possible to analyze the specific effects of each sex given our sample size. However, there were no differences in the proportion between sexes in our sample, and sex was not statistically associated with the main outcome. Then, our results can be interpreted regardless of sex. This is an observational study based on a specific cohort of participants (apparently healthy and untrained individuals from 20 to 70 years old). Therefore, causal relationships and extrapolation of our findings to other populations should be avoided. The participants’ dietary and physical activity habits were not recorded. However, all blood samples were collected after a 12-h fast and participants were untrained and not engaged in a regular exercise program. On the other hand, our results were based essentially on the commonality among various levels of evidence (correlations, MSEA, multiple linear regression, and aging metabolism breakpoint) minimizing the occurrence of metabolites occasionally associated with aging. Our result appears to be robust since the age variance explained by metabolite levels was replicated and cross-validated in a Test sample. Finally, our metabolomic analysis was based on data from two analytical platforms (1H NMR and LC-HRMS) allowing for more comprehensive coverage of the metabolome, which represents a typical limitation in most of the previously reported studies. Interestingly, although a broader number of compounds was obtained through LC-HRMS, most compounds significantly related to aging were obtained by 1H NMR, highlighting the potential of this technique for conducting studies on this topic.

## 4. Materials and Methods

### 4.1. Subjects and Study Design

Participated in this study 138 apparently healthy and untrained individuals (20–70 years old), recruited through electronic and print-based media, as well as through contacts using the Cardiovascular Physical Therapy Laboratory (LFCV) database at the Universidade Federal de São Carlos (UFSCar), São Carlos, Brazil. All participants had undergone physical exams (height, weight, and BMI measurements) and anamnesis including a detailed personal medical and disease history, history of family diseases, use of regular medications, reports about specific diets, and physical activity. All included participants were free from health conditions such as respiratory, musculoskeletal, metabolic, and neurological issues as well as from any history of cardiovascular disease; nonsmokers; non-obese (BMI < 30 kg·m^−2^); non-alcoholics or users of illicit drugs or regular medications related to chronic conditions. The cardiovascular condition of the participants was examined by a cardiologist in rest and ergometric test. All subjects who had cardiovascular alterations such as excessive arrhythmias, myocardium ischemic signals, or blood pressure hyperreactivity in the ergometric test and/or cardiopulmonary exercise test [59], as well as severe or recurrent hypotension, evident blood test alterations during the experimental protocol, were excluded. A detailed flowchart describing the recruitment process of this study is presented in Figure S2.

The study was approved by the Human Research Ethics Committee (number: 173/2011) and conducted in accordance with the standards set by the Declaration of Helsinki. All participants signed a free and informed consent form after agreeing to participate in this study. 

Resting blood samples were collected from each participant for clinical markers and metabolomic analysis. The metabolomics analysis was performed using 1H NMR and LC-HRMS.

### 4.2. Blood Sample Collection

Resting venous blood samples were collected in the fasted state (12 h) in the morning by puncture of the antecubital vein in vacuum tubes without anticoagulant by experienced professional. In addition, participants were instructed not to perform any strenuous exertion for at least two days before the blood collection, and not to consume any stimulant drink or food (such as coffee, energy drinks, chocolate, and foods with a lot of sugar), and alcoholic beverages on the day before blood collection.

For clinical markers, blood tubes were analyzed in a specialized laboratory (UNIMED Clinical Analysis Laboratory of São Carlos) to assess the participants’ health status. For metabolomic analysis, blood samples were collected in serology tubes (S-Monovette 4.9 mL, Sarstedt, Germany) and centrifuged at 1450× *g* for 10 min (Sorvall ST Benchtop Centrifuge, Thermo Scientific, Waltham, MA USA), and the supernatant serum was collected and stored at −80 °C until further analyses [60].

As the phase of the menstrual cycle, is known to impact the metabolite profile in women [61], for all women of reproductive age, blood sampling was performed between the 7th and 10th day of the menstrual cycle (follicular phase) [60]. The status of postmenopausal women was determined by the absence of menstrual bleeding for at least one year [62]. All involved women were not using contraceptives (reproductive age) or hormone replacement therapy (post-menopausal age).

### 4.3. Clinical Markers

The health status of the participants was verified by the fasting values of clinical markers, such as total cholesterol, VLDL, LDL, HDL, triacylglyceride, glucose, uric acid, urea, creatinine, and high-sensitivity C-reactive protein (hs-CRP). The total cholesterol, triacylglyceride, uric acid, urea, glucose, and creatinine were measured using wet chemistry (except for LDL that was calculated from the Friedewald equation) (Advia 1800, Siemens, Erlangen, Germany). The hs-CRP was quantified by turbidimetry (Advia 1800, Siemens, Erlangen, Germany). 

### 4.4. 1H NMR-Based Metabolomics

Serum samples (500 μL) were filtered in 3 kDa filters (Amicon Ultra) by centrifugation at 14,000× *g* for 30 min at 4 °C to macromolecule removal. Previously, filters were washed five times with 500 μL of Milli-Q water, followed by centrifugation at 14,000× *g* for 5 min at 4 °C, and spinning (filter reverse and rotation at 7500× *g* for 60 s) to eliminate any residue of Milli-Q water. The filtered sample was transferred to 5-mm NMR tubes containing phosphate buffer [(monobasic sodium phosphate, NaH_2_PO_4_, 119.97 g·mol^−1^; dibasic sodium phosphate, Na_2_HPO_4_, 141.96 g·mol^−1^), TMSP-d_4_ (3-(trimethylsilyl)-2,2′,3,3′- tetradeuteropropionic acid) at 5 mmol⋅L^−1^ as an internal reference], and D_2_O (99.9%; Sigma-Aldrich, San Luis, CA, USA) [63], with the respective proportion: 100 μL, 40 μL, and 260 μL. The final concentration of the internal reference (TMSP-d_4_) was 0.5 mmol⋅L^−1^_._ All the NMR measurements were acquired from a 14.1 Tesla Bruker spectrometer (600 MHz for hydrogen frequency), equipped with a 5 mm TCI cryoprobe at 298 K. For the 1D 1H NMR spectrum acquisition, a pulse sequence with H_2_O presaturation signal (named by Bruker as noesypr1d) was used adopting a continuous wave, assuming the following acquisition parameters: acquisition time (AQ = 3.63 s), spectral width (SW = 30 ppm), relaxation delay (d1 = 4 s), the 90º pulse time (p1 = 9.5 μs) and number of scans (ns = 128). All spectra were processed with 0.3 Hz line broadening (lb) to attenuate the noise in the spectral signals. After spectrum acquisition, baseline corrections, characterization, and quantification of metabolites present in the samples were conducted using Suite 8.6 Chenomx software (Chenomx Inc., Edmonton, AB, Canada) by the TMSP-d_4_ (0.5 mmol·L^−1^) signal as an internal reference to quantify other metabolites (Figure S3). Additionally, 2D Hetero Single Quantum Coherance (HSQC), Heteronuclear Multiple Bond Correlation (HMBC), and COrrelated SpectroscopY (COSY) experiments were used to auxiliate in the identification of the most relevant compounds initially characterized by Chenomx software (Table S1). The followed parameters were assumed for the HSQC and HMBC experiments: SWF1 238.88 ppm and SWHF2 30.03 ppm, d1 = 2 s, number of experiments in F1 = 256 and F2 = 4096, ns = 128 for HSQC and ns = 256 for HMBC; and for COSY experiment: SWF1 = 30.03 ppm and SWHF2 = 30.03 ppm, d1 = 2 s, number of experiments in F1 = 256 and F2 = 4096, and ns = 64. All data were processed using TopSpin 3.1.3 software.

### 4.5. LC-HRMS-Based Metabolomics

Serum samples, stored at −80 °C, were firstly thawed on ice and vortexed for 15 s. Afterward, the samples were submitted to a protein precipitation sample treatment. An aliquot of 150 μL of serum was transferred to a new *Eppendorf* and 450 μL of cold methanol was added to the sample to initiate the protein precipitation and metabolite extraction. The mixture was stored at −20 °C for 5 min. Then, the tubes were vortexed for 20 s and centrifuged at 7267× *g* at 4 °C for 10 min. Next, aliquots of 200 µL of the supernatant were transferred to new microtubes and 20 µL of an internal standard (5 mmol·L^−1^ of anhydrous L-Leucine-enkephalin acetate) was added to the samples and stored at −20 °C until analysis by LC-HRMS. A blank sample was prepared with 100 μL of methanol. Quality control (QC) samples were prepared from aliquots of 15 μL of the all-serum samples that had already been subjected to the protein precipitation process as described above and were injected in triplicate throughout the batch of experimental samples. 

The UHPLC Agilent system (model 1290 Infinity II, Agilent Technologies, Santa Clara, CA, USA) consisted of a binary LC-G712A pump with a blend assist G7104A, a vial sampler LC injector G7129C, and a column compartment G7129B. HyStar workstation software was used for data acquisition (HyStar v2, Bruker Daltonics, Bremen, Germany) and a Compass Data Analysis was used for data analysis and processing (DataAnalysis v3.2, Bruker Daltonics). Chromatographic analyses were performed with an Eclipse SDB-C18 column (100 × 3.0 mm i.d; 3.5 μm) (Agilent Technologies) employing a gradient elution using water + 0.1% formic acid (solvent A) and acetonitrile + 1% formic acid (solvent B) as the mobile phase at a flow rate of 0.4 mL·min^−1^ and temperature set at 40 °C. The total run time was 30 min using the following multistep gradient: 0 min, 1% B; 0–3.0 min, 1–2% B; 3–10 min, 2–30% B; 10–15 min, 30–50% B; 15–18 min, 50–80% B; 18–20 min, 80–90% B; 20–22 min, 90–95% B; 22–26 min, 95–99% B; 26.01–28 min, 99% B, for column cleaning and a conditioning cycle time of 3 min with the same initial conditions of 1% B. The injection volume was 5 μL.

The detection of compounds was performed on a quadrupole time-of-flight mass spectrometer (QqTOF), model Impact HD (Bruker Daltonics) equipped with an electrospray (ESI) interface operating in negative or positive ionization mode. Centroid acquisition mode was used for data collection and storage. The full MS and MS/MS data were acquired through Compass QtofControl v3.4 (Bruker Daltonics) and the data were processed using DataAnalysis v4.2 software (Bruker Daltonics). The ion source optimal parameters were set as follows: capillary voltage, 3600 V and 3000 V for the positive and negative ionization mode, respectively. All other parameters were the same for both ionization modes used: end plate offset, 450 V; nebulizer, 4 bar; dry heater temperature, 180 °C; dry gas flow, 8 L·min^−1^; quadrupole ion energy, 5 eV, and full-MS scan range, *m*/*z* 50–1300.

A dynamic stepping was used for data-dependent acquisition (DDA) MS/MS mode where the collision RF was set to vary between 200.0 to 550.0% Vpp; the transfer time was set to vary 50.0 to 90.0 µs; with 50.0% timing each. The collision energy for the ion fragmentation was programmed to vary from 100 to 250.0% from 20 eV initially set, with the following isolation mass: *m*/*z* 100, 200, and 300:4 width; for *m*/*z* 700 and 1000:6 width. Funnels RF 1 and 2 were 250.0 and 150.0 Vpp, respectively. The hexapole RF was 50.0 Vpp, the quadrupole ion energy was 5.0 eV with a pre pulse storage of 6.0 µs. Quadrupole ion energy and collision cell energy were both set at 5 eV. The parameters used to trigger the MS/MS fragmentation were 2.0 Hz for low counts (10,000 cts/per 1000 sum) and 4.0 Hz for high counts (100,000 counts/per 1000 sum), using a total cycle time range of 3 s; absolute threshold of 1491 counts (302 counts/per 1000 sum), active exclusion 1 spectra; release after 0.90 min, while the full MS acquisition was set at 2.0 Hz. Internal mass spectrometer calibration was performed with 1 mmol·L^−1^ of sodium formate prepared in acetonitrile, using a quadratic high-precision calibration (HPC) regression mode. The calibration solution was injected at the end of each analytical run, and all the spectra were recalibrated before compound identification.

Bruker Profile Analysis v2.1 software (Bruker Daltonics) was used to process the LC-HRMS data. The bucket generation was performed with the following parameters: S/N threshold = 2; correlation coefficient threshold = 0.2; minimum compound length = 10 spectra; smoothing width = 1. All features detected by the LC-MS were subjected to data processing consisting of the inclusion of features based on values greater than 5% from blank samples; coefficient of variation (CV%) of QCs samples (mean of replicates) lower than 20%; missing data lower than 10% in experimental samples. The remaining features were normalized by non-linear local regression (LOESS) to account for the instrumental stability using the Noreva 2.0 software (Figure S4) [64].

Data Analysis v4.2 (Bruker Daltonics) was used to perform the identification of the fragment ions (MS/MS) of those detected compounds, which were further putatively confirmed by comparing their fragment ions with those data in the HMDB (https://hmdb.ca (accessed on 26 April 2022)), Mass Bank (https://massbank.eu/MassBank/ (accessed on 26 April 2022)), CEU Mass Mediator (http://ceumass.eps.uspceu.es/ (accessed on 26 April 2022)) databases. Compounds were identifyed based on protonated, desprotonated, and adduct ions as following: [M + H]^+^, [M + H-2H_2_O]^+^, [M + H-H_2_O]^+^, [M + NH_4_-H_2_O]^+^, [M + NH_4_]^+^, [M + Na]^+^, [M + CH_3_OH + H]^+^, [M + K]^+^, [M + ACN + H]^+^, [M + 2Na-H]^+^, [M + IsoProp + H]^+^, [M + ACN + Na]^+^, [M + 2K-H]^+^, [M + 2ACN + H]^+^, [M + IsoProp + Na + H]^+^, [M + H + HCOONa]^+^, [2M + H]^+^, [2M + NH_4_]^+^, [2M + Na]^+^, [2M + 2H + 3H2O]^+^, [2M + K]^+^, [2M + ACN + H]^+^, [2M + ACN + Na]^+^, [2M + H-H2O]^+^, [M + 2H]^+^, [M + H + NH_4_]^+^, [M + H + Na]^+^, [M + H + K]^+^, [M + ACN + 2H]^+^, [M + 2Na]^+^, [M + H + Na]^+^, [M + 2ACN + 2H]^+^, [M + 3ACN + 2H]^+^, [M + 3H]^+^, [M + 2H + Na]^+^, [M + H + 2Na]^+^, [M + 3Na]^+^, and [M + H + 2K]^+^ for the positive ionization mode; and [M-H]^−^, [M-H_2_O-H]^−^, [M-Na-2H]^−^, [M + Cl]^−^, [M + K-2H]^−^, [M-FA-H]^−^, [M-Hac-H]^−^, [M-TFA-H]^−^, [M-H + HCOONa]^−^, [2M-H]^−^, [2M + FA-H]^−^, [2M + Hac-H]^−^, [3M-H]^−^, and [M-3H]^−^ for the negative ionization mode.

### 4.6. Statistical Analysis

Multiple imputations were conducted for missing values of compounds and sample characterization variables (except age), using the Markov Chain Monte Carlo (MCMC) approach for nonmonotone missing data [65]. The median and maximum number of missing values per variable within the entire data set was two (interquartile range: 0–3) and 13, respectively. The total number of missing values within the entire data set was <2%. 

For all continuous variables, the data distributions were checked using the Shapiro-Wilk test. To improve normality of distributions, all variables were Box-Cox transformed for subsequent analyses [66]. However, all transformed data were presented in their original scale for easier interpretation. A Student’s t-test for independent samples and a Chi-square test were used to compare physical and clinical characteristics of the participants between Training and Test samples.

To investigate the aging-related serum metabolic profile, firstly linear regression including physical characteristics of the participants (BMI and sex) as independent variables and age as the dependent variable were performed. After, partial correlations using Pearson correlation coefficients were run to determine the relationship of the clinical markers and serum metabolite levels with age whilst controlling for BMI. For these hypothesis-generating analyses, we used the Benjamini-Hochberg procedure to account for multiple tests and employed a FDR of 0.2 and a significance level threshold at a nominal value of *p* < 0.05 to determine statistical significance [67].

For the identification of aging-regulated metabolic pathways, based on all correlational analyses performed, clinical markers and metabolites that showed significant correlation were listed for a Metabolite Set Enrichment Analysis (MSEA) based on normal human metabolic pathways [The Small Molecule Pathway Database (SMPD) library] using an algorithm for Over Representation Analysis and Hypergeometric test to evaluate whether a particular metabolite set is represented more than expected by chance within the given compound list [68]. Additionally, the network of the most enriched pathways enjoying connections was displayed. These analyzes were performed using the web-based tool MetaboAnalyst 5.0 (https://www.metaboanalyst.ca/ (accessed on 23 May 2022)).

To determine the overall contribution of the serum metabolic levels on the aging process, metabolites significantly retained in the previous two steps (correlation and MSEA) were analyzed in a multiple linear regression model with forward stepwise selection. This approach allowed select metabolites with statistical and biological relevance, minimizing the occurrence of metabolites occasionally associated with the phenotype in final model [69,70]. Prior to this analysis, the data set was randomly split into a Training set (*n* = 105, ~75% of the cohort) that was used for the main analysis and an independent Test set (*n* = 33, ~25% of the cohort) for cross-validation. Metabolite levels as well as BMI (covariate) were Box-Cox transformed and standardized to mean = 0 and multiples of one standard deviation. For Box-Cox transformations, the lambda value was optimized for each variable, being selected the lambda value in a range of −10 to 10 that resulted in the smallest standard deviation. For both Training and Testing samples, the same optimized lambda value was applied. Scaling in the Test sample was applied using the same mean and standard deviation used to scaling the Training sample [71]. The assumption of multicollinearity of measures between the independent variables was assessed by the variance inflation factor (VIF~1) and the normality of residue distribution was confirmed by visual inspection of the frequency histograms. 

Afterward, metabolites retained in the correlation and MSEA steps also were analyzed in a PCA to extract a score representative of the major factor of variability in the metabolism weighted for each metabolite and BMI. PCA also was cross-validated using Training and Test samples. Then, the scores from the first principal component (PC1), representing a major portion of the variability in metabolism for each individual were plotted as a function of age to identify a metabolism breakpoint. Each point on the graph corresponds to a representation of metabolism at a given age for each individual. The total points were divided into two sets of points. The first 35 points, representing one-third of the sample, were arbitrarily included in the first set, with the remaining points included in a second set. After that, a straight regression line (PC1 scores vs. age) was fitted to each set of points, and the coefficient of determination (r^2^) from each line was calculated. The product of these two coefficients, which represent the linearity of the two sets of points, resulted in an index and was recorded for further analysis. The initial value of the second set of points was included in the first set of points, increasing the number of points in the first set and decreasing the number of points in the second set. New regression lines were plotted, and the corresponding regression coefficients calculated, resulting in a new index for two new sets of points. This procedure was repeated, and at each time incorporated the next value of the second set of points into the first set of points, until the second set was composed of the last 35 points of the file (one-third of the sample) and the first set with the remainder of the points. The two sets of points that elicited the pair of lines with the largest product of the two coefficients of determination were chosen and the intersection between these lines was identified and defined as the metabolism breakpoint with aging [72,73]. Figure 3B shows the metabolism breakpoint procedure.

All the analyses described above (comparisons between groups, correlations, linear regressions, and PCAs), were performed using SPSS 25.0 software (Chicago, IL, USA). The level of significance adopted was 5%.

## 5. Conclusions

The aging process is potentially associated with an increased flux of the branched-chain amino acids (valine, leucine, and isoleucine) degradation pathway, especially after the third decade of life, which is accompanied by a decrease in the urea cycle’s ability to excrete its degradation by-products, evidenced by a progressive increase in serum levels of ornithine and aspartate. 

## Figures and Tables

**Table 1:** Age, physical, and clinical characteristics of the participants (*n* = 138). Data are median (interquartile range).

Variables	Training Sample (*n* = 105)		Test Sample (*n* = 33)		*p*-Value ^#^
Age (years)	42.0	(30.0–51.5)	40.0	(29.0–53.0)	0.887
Height (m)	1.69	(1.62–1.76)	1.65	(1.62–1.77)	0.467
Body mass (kg)	70.4	(63.1–80.0)	68.4	(58.6–80.0)	0.485
BMI (km·m^−2^)	24.8	(22.9–26.9)	24.9	(21.7–26.6)	0.873
Total cholesterol (mg·dL^−1^)	188.0	(165.0–204.0)	184.0	(163.0–203.0)	0.454
HDL (mg·dL^−1^)	52.0	(43.5–63.0)	57.0	(44.0–66.5)	0.455
LDL (mg·dL^−1^)	113.0	(93.0–130)	103.0	(90.5–120.0)	0.177
VLDL (mg·dL^−1^)	19.0	(14.0–24.5)	15.0	(13.5–28.0)	0.974
Triacylglyceride (mg·dL^−1^)	93.0	(69.0–122.5)	77.0	(67.5–138.5)	0.998
Uric acid (mg·dL^−1^)	5.10	(4.35–6.10)	5.20	(4.3–6.40)	0.851
Creatinine (mg·dL^−1^)	0.88	(0.76–1.00)	0.94	(0.78–1.02)	0.417
Glucose (mg·dL^−1^)	90.8	(86.0–94.0)	94.0	(86.5–97.5)	0.110
Urea (mg·dL^−1^)	31.0	(27.0–37.0)	32.0	(25.5–34.5)	0.367
hs-CRP (mg·dL^−1^)	0.62	(0.18–1.22)	0.37	(0.14–1.29)	0.307

Body mass index (BMI); High-density lipoprotein (HDL); High-sensitivity C-reactive protein (hs-CRP); Low-density lipoprotein (LDL); Very low-density lipoprotein (VLDL). ^#^ *p*-values obtained by Student’s *t*-test for independent samples.

## Data Availability

All data used to support the findings of this study are included within the article and they are also available from the corresponding author upon request. Samples of the compounds are not available from the authors.

## Supplementary Materials

The following supporting information can be downloaded at: https://www.mdpi.com/article/10.3390/molecules27248656/s1. Figure S1: Scatter plots of the partial correlations (adjusted by body mass index—BMI) between age and metabolites levels (main findings). Metabolite levels were Box-Cox transformed and standardized to mean = 0 and multiples of one standard deviation (z-score). Blue and yellow colors represent low and high BMI values (z-score); Figure S2: Flowchart describing the recruitment process; Figure S3: 1H NMR spectrum with the main aging-associated serum metabolites highlighted in red, 14.1 T, 25 °C in D_2_O. TMSP-d_4_ was used as internal reference; Figure S4: Instrumental stability assessed through quality control samples for data obtained by LC-HRMS in positive and negative ionization mode; Table S1: Chemical structure and chemical shift (δ-ppm) of metabolites significantly associated with aging obtained by 1H NMR-based metabolomics; Table S2: Identification parameters of the compounds significantly associated with aging obtained by LC-HRMS-based metabolomics; Table S3. Correlations between clinical markers and age; Table S4. Correlations between obtained compounds by LC-HRMS and age; Table S5. Correlations between obtained compounds by 1H NMR and age; Table S6. Summary of the metabolic pathways associated with aging after Metabolite Set Enrichment Analysis.
