# Supplementary material for: The Aging Process: A Metabolomics Perspective
Source: Molecules. 2022 Dec 7;27(24):8656. doi: 10.3390/molecules27248656 (PMC9785117; doi:10.3390/molecules27248656)
Supplement: Supplementary file 1 [file molecules-27-08656-s001.zip › molecules-1969341-Supplementary.pdf]

The Aging Process: A Metabolomics Perspective

Alex Castro [1,*], Étore F. Signini [2], Juliana Magalhães De Oliveira [1], Maria Carolina Bezerra Di Medeiros Leal [1],
Patrícia Rehder-Santos [2], Juliana C. Millan-Mattos [2], Vinicius Minatel [2], Camila B. F. Pantoni [2], Regina V. Oliveira [1], Aparecida M. Catai [2] and Antônio G. Ferreira [1,*]

[1] Department of Chemistry, Universidade Federal de São Carlos, 13565-905 São Carlos, Brazil
[2] Department of Physiotherapy, Universidade Federal de São Carlos, 13565-905 São Carlos, Brazil
* Correspondence: ax.castro@yahoo.com.br (A.C.); giba@ufscar.br (A.G.F.).

Supplementary Material

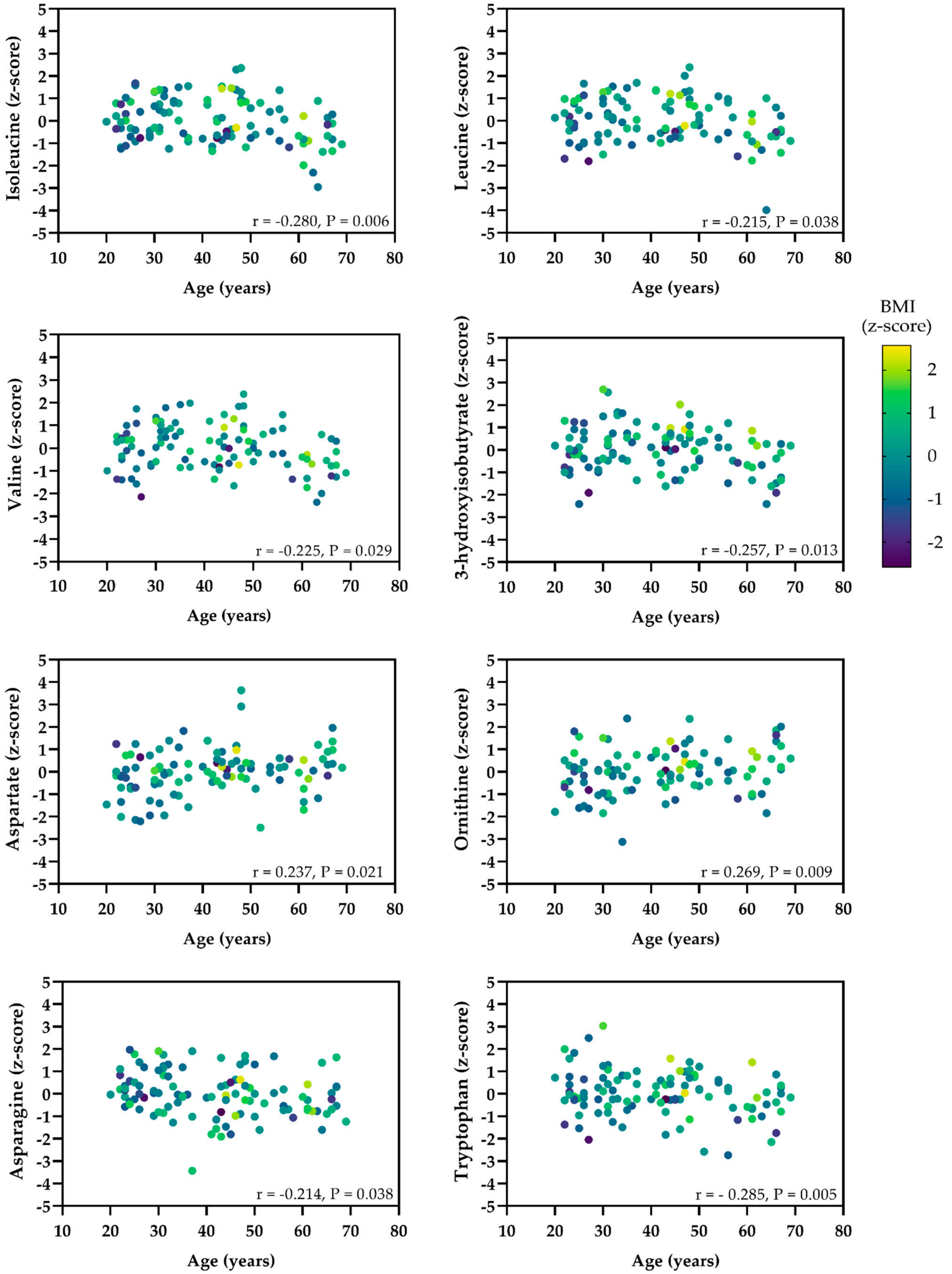

Figure S1. Scatter plots of the partial correlations (adjusted by body mass index - BMI) between age and metabolites levels (main findings). Metabolite levels were Box-Cox transformed and standardized to mean = 0 and multiples of 1 standard deviation (z-score). Blue and yellow colors represent low and high BMI values (z-score).

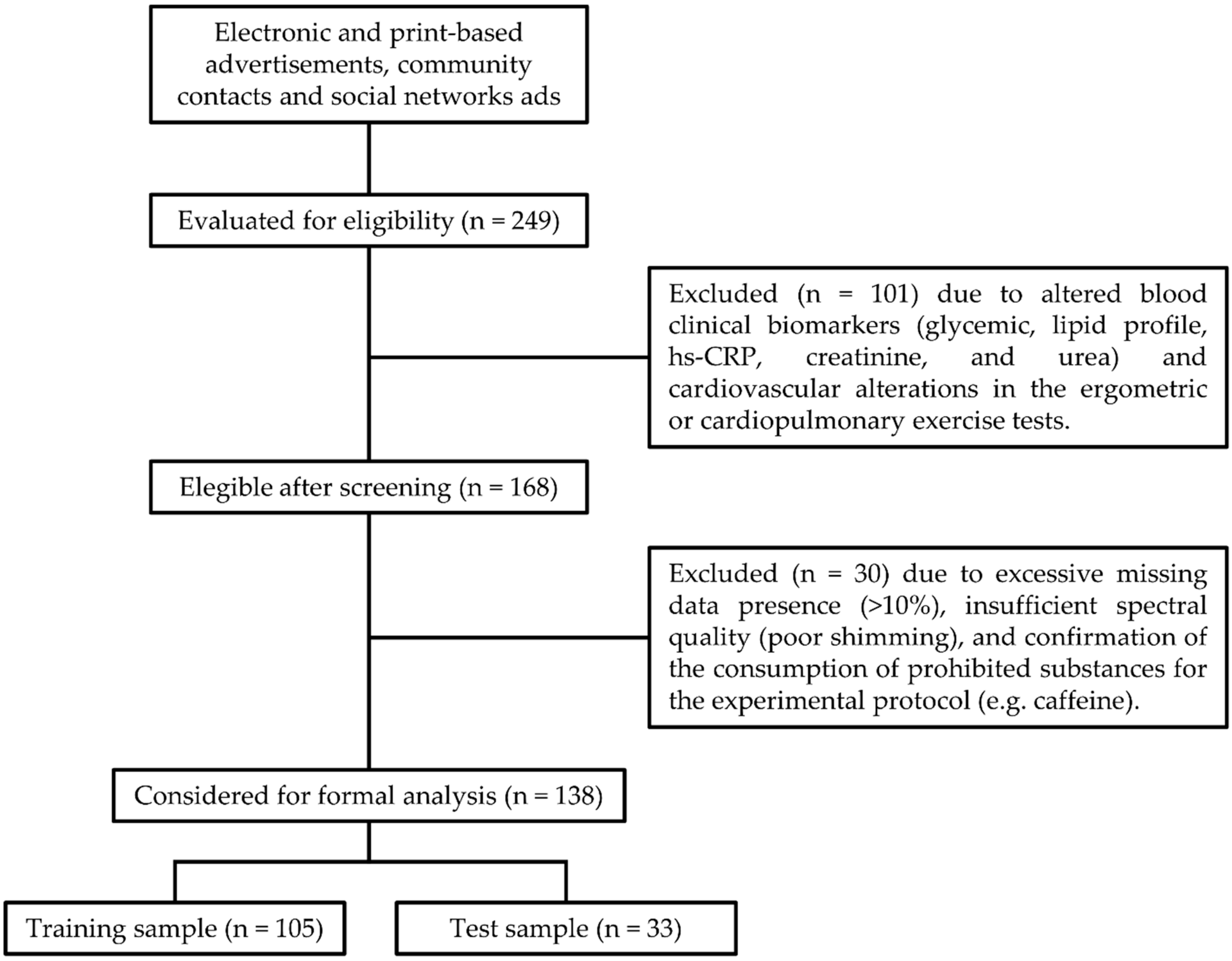

Figure S2. Flowchart describing the recruitment process of individuals.

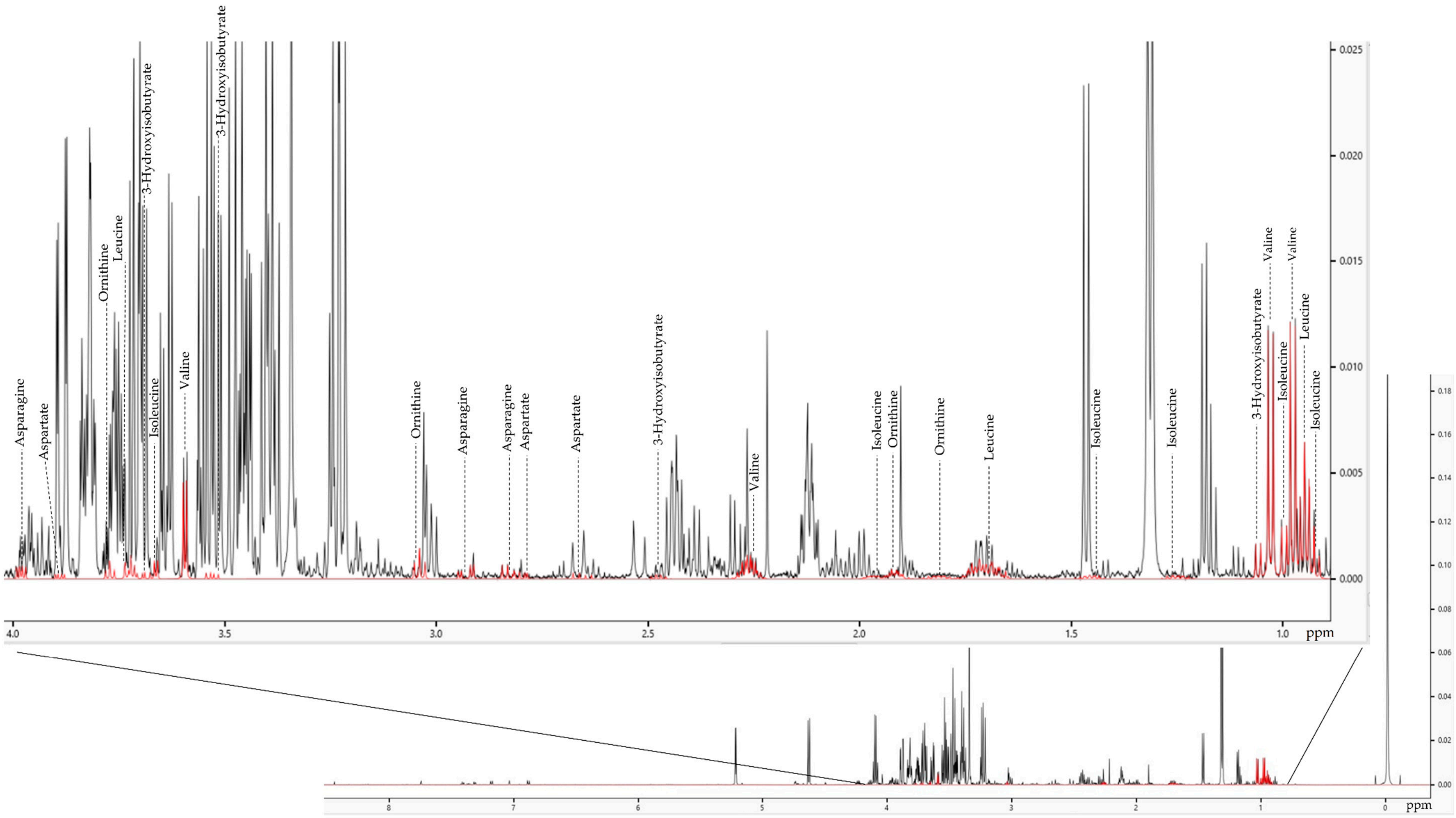

Figure S3. 1H NMR spectrum with the main aging-associated serum metabolites highlighted in red, 14.1 T, 25°C in D_2O. TMSP-d_4 was used as internal reference.

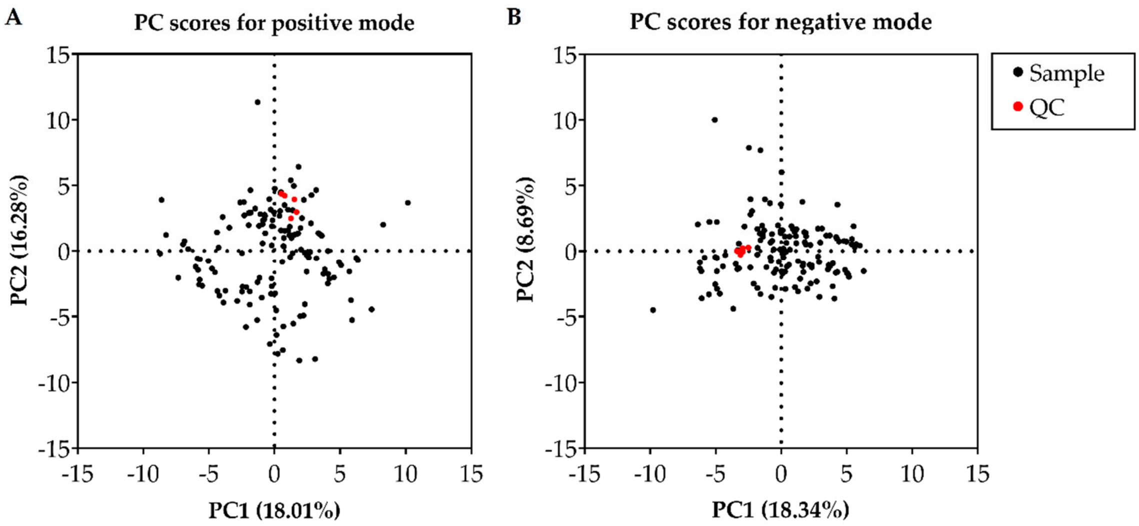

Figure S4. Instrumental stability assessed through quality control samples for data obtained by LC-HRMS in positive and negative ionization mode.

Table S1. Chemical structure and chemical shift (δ - ppm) of compounds significantly associated with aging obtained by 1H NMR-based metabolomics.

Nº	Compounds and Structure	δ ^{1}H ppm (Multiplicity*, Nº H, J)	COSY ^{1}H/^{1}H	HSQC ^{13}C/^{1}H	HMBC
I	Aspartate<br>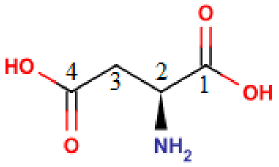	**1.** ---<br>**2.** 3.88-3.90 (m – 1H)<br>**3.** 2.65 (dd – 2H, 17.5 Hz, 8.88 Hz)<br>**3.** 2.80 (dd – 2H, 17.5 Hz, 3.64 Hz)<br>**4.** ---	**2.** 3.88-3.90/2.65<br>**2.** 3.88-3.90/2.80	Unobserved	**3.** 182.0
II	Isoleucine<br>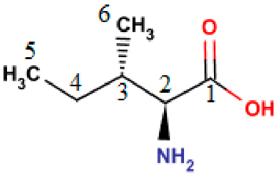	**1.** ---<br>**2.** 3.66 (d – 1H)<br>**3.** 1.94-2.00 (m – 1H)<br>**4.** 1.24-1.45 (m – 2H)<br>**5.** 0.92 (t – 3H)<br>**6.** 0.99 (d – 3H, 7.02 Hz)	**3.** 1.94-2.00/1.45<br>**5.** 0.92/1.45<br>**6.** 0.99/1.94-2.00	**2.** 62.7/3.66<br>**5.** 13.8/0.92<br>**6.** 17.6/0.99	**5.** 27.7<br>**6.** 27.7; 38.9; 62.7
III	Leucine<br>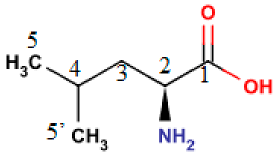	**1.** ---<br>**2.** 3.71-3.73 (m – 1H)<br>**3.** 1.65-1.75 (m –2H)<br>**4.** 1.65-1.75 (m – 1H)<br>**5.** 0.94 (d – 3H, 6.60 Hz)<br>**5′.** 0.95 (d – 3H, 6.60 Hz)	**2.** 3.71-3.73/1.72<br>**5.** 0.94/1.69<br>**5′.** 0.95/1.69	**2.** 56.2/3.71-3.73<br>**3.** 27.0/1.65-1.75<br>**4.** 42.8/1.65-1.75<br>**5.** 24.7/0.94<br>**5′.** 24.7/0.95	3. 42.8<br>**5.** 24.7; 27.0; 42.8<br>**5′.** 24.7; 27.0; 42.8
IV	3-Hydroxyisobutyrate<br>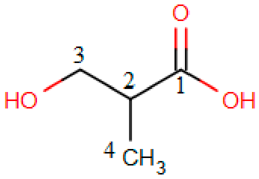	**1.** ---<br>**2.** 2.44-2.50 (m – 1H)<br>**3.** 3.51-3.55 (m – 2H)<br>**4.** 1.06 (d – 3H, 7.15 Hz)	**2.** 2.44-2.50/1.06	**3.** 3.51-3.55/65.5<br>**4.** 1.06/19.1	3. 175.4
V	Valine<br>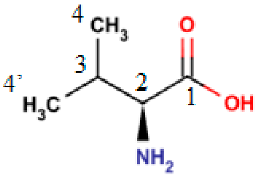	**1.** ---<br>**2.** 3.59 (d – 1H, 4.50 Hz)<br>**3.** 2.22-2.30 (m)<br>**4.** 0.97 (d – 3H, 7.00 Hz)<br>**4′.** 1.03 (d – 3H, 7.00 Hz)	**4.** 0.97/2.26<br>**4′.** 1.03/2.26	**2.** 63.3/3.59<br>**3.** 31.3/2.22-2.30<br>**4.** 19.7/0.97<br>**4′** 20.9/1.03	**4.** 20.9; 31.3; 63.3<br>**4′.** 19.7; 31.3; 63.3
VI	Ornithine<br>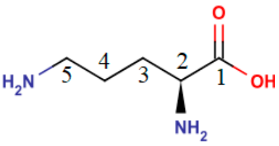	**1.** ---<br>**2.** 3.76-3.78 (m – 1H)<br>**3.** 1.88-1.97 (m – 2H)<br>**4.** 1.70-1.84 (m – 2H)<br>**5.** 3.04 (t – 2H, 7.46 Hz; 14.93 Hz)	**2.** 3.76-3.78/1.88-1.97<br>**3.** 1.88-1.97/3.76-3.78<br>**4.** 1.70-1.84/3.04<br>**5.** 3.04/1.82	**3.** 1.88-1.97/30.5<br>**5.** 3.04/41.9	Unobserved
VII	Asparagine<br>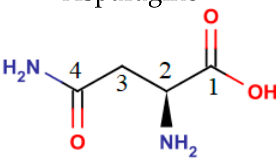	**1.** ---<br>**2.** 3.97-3.99 (m – 1H)<br>**3.** 2.85 (dd – 1H, 4.2 Hz, 16.9 Hz)<br>**3.** 2.95 (dd – 1H, 8.0 Hz, 16.9 Hz)<br>**4.** ---	**3.** 2.85/3.99	**2.** 3.97-3.99/57.9	Unobserved

singlet (s); doublet (d); triplet (t); multiplet (m).

Table S2. Identification parameters of the compounds significantly associated (P < 0.05 and < FDR) with aging obtained by LC-HRMS-based metabolomics.

Compound	HMDB code	Formula	Monoisotopic Mass	Ion	*m/z*	Matched fragment ions (ppm error)
Tryptophan	HMDB0030396	$C_{11}H_{12}N_2O_2$	204.0898	M-H-	203.0825	74.0243 (1.3 ppm); 116.0502 (1.5 ppm); 142.0663 (4.4 ppm); 159.0933 (6.7 ppm); 186.0563 (4.2 ppm); 203.0825 (2.2 ppm).
Unknown	-	-	-	Negative	129.0558	-
Unknown	-	-	-	Negative	537.4146	-
Unknown	-	-	-	Negative	130.0886	-

FDR: False discovery rate.

Table S3. Correlations between clinical markers and age.

Clinical markers	Correlation coefficient (r)	P-value	FDR	Decision
Total cholesterol	0.37	0.0002	0.0200	SIG
LDL	0.24	0.0184	0.0400	SIG
HDL	0.22	0.0343	0.0600	SIG
VLDL	0.21	0.0388	0.0800	SIG
Glucose	0.21	0.0394	0.1000	SIG
Triacylglyceride	0.21	0.0404	0.1200	SIG
Ureia	0.19	0.0639	0.1400	SIG
hs-CRP	0.15	0.1632	0.1600	NS
Creatinine	0.07	0.5033	0.1800	NS
Uric acid	-0.01	0.9149	0.2000	NS

FDR: False discovery rate. SIG: P < 0.05 and < FDR; NS: P > 0.05 and/or > FDR.

Table S4. Correlations between obtained compounds by LC-HRMS and age.

Compounds [retention time (s):mass-to-charge ratio (m/z)]	Ionization mode	Correlation coefficient (r)	P-value	FDR	Decision
815.1 : 129.056	Negative	0.47	0.0000	0.0016	SIG
1340.5 : 537.416	Negative	-0.34	0.0009	0.0031	SIG
1351.1 : 467.374	Negative	-0.29	0.0053	0.0047	NS
511.3 : 203.083	Negative	-0.29	0.0053	0.0063	SIG
663.0 : 178.051	Negative	0.27	0.0079	0.0078	NS
141.7 : 130.087	Negative	0.27	0.0079	0.0094	SIG
942.5 : 1086.177	Positive	0.25	0.0162	0.0109	NS
1232.8 : 586.314	Negative	0.24	0.0179	0.0125	NS
1445.7 : 593.478	Negative	-0.24	0.0215	0.0141	NS
853.5 : 619.751	Positive	0.23	0.0277	0.0156	NS
854.0 : 617.738	Negative	0.21	0.0413	0.0172	NS
803.9 : 655.280	Positive	0.21	0.0464	0.0188	NS
1294.9 : 746.442	Positive	-0.20	0.0530	0.0203	NS
1395.1 : 868.598	Positive	0.19	0.0615	0.0219	NS
808.9 : 653.267	Negative	0.19	0.0653	0.0234	NS
720.7 : 195.088	Positive	0.19	0.0656	0.0250	NS
617.1 : 942.343	Negative	0.18	0.0865	0.0266	NS
1404.2 : 652.499	Positive	0.18	0.0884	0.0281	NS
1413.5 : 916.656	Positive	0.18	0.0900	0.0297	NS
854.1 : 1236.480	Negative	0.16	0.1145	0.0313	NS
1186.9 : 802.560	Negative	-0.16	0.1227	0.0328	NS
73.1 : 118.087	Positive	-0.16	0.1292	0.0344	NS
1045.0 : 612.330	Negative	-0.15	0.1424	0.0359	NS
92.9 : 167.021	Negative	-0.15	0.1465	0.0375	NS
1066.4 : 415.211	Positive	-0.14	0.1673	0.0391	NS
1302.9 : 599.381	Positive	-0.14	0.1685	0.0406	NS
120.1 : 191.020	Negative	0.14	0.1828	0.0422	NS
1099.3 : 380.255	Positive	0.14	0.1910	0.0438	NS
1056.9 : 568.361	Negative	-0.13	0.2105	0.0453	NS
1403.2 : 564.446	Positive	0.13	0.2207	0.0469	NS
136.3 : 180.067	Negative	-0.13	0.2274	0.0484	NS
1427.1 : 853.522	Positive	-0.12	0.2323	0.0500	NS
1373.3 : 281.248	Negative	0.12	0.2479	0.0516	NS
1364.0 : 833.505	Positive	-0.12	0.2554	0.0531	NS
1000.5 : 228.196	Positive	-0.12	0.2576	0.0547	NS
1411.1 : 872.630	Positive	0.12	0.2652	0.0563	NS
89.9 : 162.839	Negative	0.11	0.2840	0.0578	NS
386.5 : 117.056	Negative	-0.11	0.2997	0.0594	NS
1301.2 : 708.416	Positive	0.11	0.3053	0.0609	NS
1076.4 : 183.080	Positive	-0.10	0.3228	0.0625	NS
1405.0 : 696.525	Positive	0.10	0.3228	0.0641	NS
1373.5 : 381.174	Negative	0.10	0.3355	0.0656	NS
270.0 : 164.072	Negative	0.10	0.3362	0.0672	NS
1145.2 : 500.278	Negative	0.10	0.3396	0.0688	NS
912.6 : 119.086	Positive	-0.10	0.3518	0.0703	NS

797.6 : 173.082	Negative	-0.09	0.3696	0.0719	NS
941.4 : 1102.185	Positive	-0.09	0.3895	0.0734	NS
1059.0 : 452.203	Positive	-0.09	0.3903	0.0750	NS
460.7 : 145.051	Negative	-0.09	0.3940	0.0766	NS
1043.5 : 538.315	Negative	-0.09	0.3944	0.0781	NS
1381.3 : 686.444	Positive	0.08	0.4328	0.0797	NS
1283.1 : 686.358	Positive	-0.08	0.4408	0.0813	NS
1043.8 : 484.228	Positive	0.08	0.4451	0.0828	NS
1402.5 : 395.275	Negative	0.08	0.4548	0.0844	NS
1445.1 : 310.310	Positive	-0.08	0.4554	0.0859	NS
912.6 : 147.080	Positive	-0.08	0.4616	0.0875	NS
1090.5 : 540.331	Negative	-0.08	0.4703	0.0891	NS
1143.7 : 614.346	Negative	-0.07	0.4863	0.0906	NS
1303.7 : 279.233	Negative	0.07	0.4881	0.0922	NS
1406.6 : 100.933	Negative	0.07	0.4912	0.0938	NS
1159.3 : 476.278	Negative	0.07	0.4971	0.0953	NS
1035.8 : 293.176	Negative	0.07	0.5095	0.0969	NS
1482.6 : 773.507	Positive	-0.07	0.5256	0.0984	NS
1316.9 : 775.463	Positive	0.07	0.5288	0.1000	NS
778.8 : 516.301	Positive	-0.07	0.5290	0.1016	NS
1443.2 : 744.485	Positive	-0.07	0.5331	0.1031	NS
842.1 : 696.773	Negative	-0.06	0.5374	0.1047	NS
1286.6 : 303.233	Negative	0.06	0.5619	0.1063	NS
1444.2 : 284.295	Positive	0.06	0.5765	0.1078	NS
772.0 : 731.317	Negative	-0.06	0.5804	0.1094	NS
1464.2 : 283.264	Negative	0.06	0.5972	0.1109	NS
1323.5 : 766.458	Positive	-0.05	0.6034	0.1125	NS
716.9 : 265.118	Positive	0.05	0.6078	0.1141	NS
1396.9 : 824.501	Positive	-0.05	0.6159	0.1156	NS
64.7 : 145.062	Negative	-0.05	0.6207	0.1172	NS
1016.4 : 187.007	Negative	0.05	0.6254	0.1188	NS
1400.2 : 350.211	Positive	-0.05	0.6279	0.1203	NS
87.1 : 89.024	Negative	0.05	0.6293	0.1219	NS
156.5 : 103.040	Negative	-0.05	0.6314	0.1234	NS
1330.3 : 282.280	Positive	0.05	0.6342	0.1250	NS
1119.9 : 636.260	Negative	-0.05	0.6458	0.1266	NS
925.2 : 583.256	Negative	-0.05	0.6618	0.1281	NS
839.4 : 663.266	Positive	0.05	0.6635	0.1297	NS
772.7 : 432.280	Positive	-0.04	0.6749	0.1313	NS
52.3 : 158.978	Negative	0.04	0.6762	0.1328	NS
1429.2 : 359.315	Positive	-0.04	0.6791	0.1344	NS
840.6 : 698.786	Positive	-0.04	0.6846	0.1359	NS
106.0 : 169.036	Positive	-0.04	0.6898	0.1375	NS
758.8 : 388.254	Positive	0.04	0.6986	0.1391	NS
847.6 : 187.097	Negative	-0.04	0.7010	0.1406	NS
788.6 : 560.327	Positive	0.04	0.7043	0.1422	NS
1353.5 : 657.423	Positive	-0.04	0.7072	0.1438	NS
1465.0 : 383.189	Negative	-0.04	0.7123	0.1453	NS
1264.2 : 271.228	Negative	0.04	0.7292	0.1469	NS

1347.0 : 795.479	Positive	-0.04	0.7315	0.1484	NS
1186.7 : 298.274	Positive	-0.04	0.7322	0.1500	NS
1320.0 : 256.264	Positive	0.03	0.7465	0.1516	NS
1322.2 : 737.437	Positive	0.03	0.7495	0.1531	NS
1345.7 : 766.459	Positive	-0.03	0.7528	0.1547	NS
1011.6 : 158.154	Positive	-0.03	0.7742	0.1563	NS
89.6 : 164.836	Negative	0.03	0.7852	0.1578	NS
1371.2 : 824.500	Positive	0.03	0.7892	0.1594	NS
1363.1 : 255.233	Negative	0.03	0.7911	0.1609	NS
1410.7 : 144.923	Negative	0.03	0.8088	0.1625	NS
767.3 : 603.791	Positive	-0.02	0.8178	0.1641	NS
1032.2 : 452.203	Positive	0.02	0.8227	0.1656	NS
1086.4 : 532.300	Negative	0.02	0.8264	0.1672	NS
809.8 : 692.405	Positive	-0.02	0.8278	0.1688	NS
1398.8 : 853.521	Positive	0.02	0.8344	0.1703	NS
1394.0 : 384.346	Positive	-0.02	0.8355	0.1719	NS
1326.0 : 628.403	Positive	-0.02	0.8427	0.1734	NS
117.3 : 199.805	Negative	-0.02	0.8497	0.1750	NS
1369.7 : 795.480	Positive	0.02	0.8498	0.1766	NS
1469.4 : 583.256	Negative	0.02	0.8602	0.1781	NS
797.6 : 604.353	Positive	-0.02	0.8694	0.1797	NS
1020.8 : 426.186	Positive	0.02	0.8706	0.1813	NS
799.9 : 702.510	Positive	0.02	0.8735	0.1828	NS
1362.9 : 355.158	Negative	-0.01	0.8894	0.1844	NS
840.6 : 717.758	Positive	-0.01	0.8959	0.1859	NS
119.9 : 195.811	Negative	0.01	0.9227	0.1875	NS
1310.5 : 379.158	Negative	-0.01	0.9268	0.1891	NS
1102.1 : 378.241	Negative	0.01	0.9294	0.1906	NS
1031.8 : 161.096	Positive	0.01	0.9336	0.1922	NS
59.0 : 112.986	Negative	-0.01	0.9344	0.1938	NS
1141.6 : 426.357	Positive	-0.01	0.9370	0.1953	NS
803.4 : 648.379	Positive	0.01	0.9383	0.1969	NS
115.6 : 128.035	Negative	0.01	0.9612	0.1984	NS
1307.1 : 337.166	Positive	0.00	0.9936	0.2000	NS

FDR: False discovery rate; SIG: P < 0.05 and < FDR; NS: P > 0.05 and/or > FDR.

Table S5. Correlations between obtained compounds by 1H NMR and age.

Compounds*	1H chemical shift (δ - ppm) used as reference for quantification	Correlation coefficient (r)	P-value	FDR	Decision
Threonine	4.23	-0.281	0.00615	0.00435	NS
Isoleucine	0.99	-0.280	0.00636	0.00870	SIG
Ornithine	3.04	0.269	0.00876	0.01304	SIG
3-Hydroxyisobutyrate	1.05	-0.257	0.01251	0.01739	SIG
Aspartate	2.66	0.237	0.02143	0.02174	SIG
Succinate	2.39	0.226	0.02880	0.02609	NS
Valine	0.97	0.225	0.02892	0.03043	SIG
Dimethylsulfone	3.13	0.217	0.03584	0.03478	NS
Leucine	0.95	-0.215	0.03754	0.03913	SIG
Asparagine	2.92	-0.214	0.03793	0.04348	SIG
Acetoacetate	2.26	-0.189	0.06755	0.04783	NS
Choline	3.19	0.173	0.09512	0.05217	NS
Glycine	3.54	0.164	0.11422	0.05652	NS
Lactate	1.31	0.155	0.13561	0.06087	NS
Histidine	7.74	-0.147	0.15693	0.06522	NS
Methionine	2.63	-0.146	0.16032	0.06957	NS
Sarcosine	2.72	0.142	0.17257	0.07391	NS
Alanine	1.46	-0.127	0.22249	0.07826	NS
Anserine	6.97	-0.127	0.22249	0.08261	NS
Glucose	5.22	0.119	0.25292	0.08696	NS
2-Hydroxyisovalerate	0.82	-0.114	0.27285	0.09130	NS
O-Acetylcarnitine	3.18	0.108	0.30160	0.09565	NS
Glutamine	2.46	-0.098	0.34895	0.10000	NS
3-Methyl-2-oxovalerate	0.88	0.096	0.35938	0.10435	NS
Ascorbate	4.49	0.095	0.35993	0.10870	NS
Citrate	2.68	0.095	0.36189	0.11304	NS
Formate	8.44	0.084	0.42155	0.11739	NS
Serine	3.94	-0.079	0.45086	0.12174	NS
3-Hydroxybutyrate	1.18	0.078	0.45212	0.12609	NS
Lysine	3.01	0.076	0.46494	0.13043	NS
N,N-Dimethylglycine	2.91	0.073	0.48235	0.13478	NS
2-Oxoisocaproate	0.92	-0.069	0.50582	0.13913	NS
2-Oxoglutarate	2.99	-0.068	0.51264	0.14348	NS
Creatinine	3.03	-0.065	0.53471	0.14783	NS
Tyrosine	6.88	0.061	0.56182	0.15217	NS
Acetate	1.9	-0.051	0.62723	0.15652	NS
2-Hydroxybutyrate	0.88	-0.034	0.74240	0.16087	NS
Glycerol	3.55	-0.026	0.80308	0.16522	NS
Proline	2.06	0.025	0.80902	0.16957	NS
Betaine	3.25	-0.016	0.87928	0.17391	NS
Hypoxanthine	8.17	0.015	0.88626	0.17826	NS
Phenylalanine	7.42	-0.011	0.91870	0.18261	NS
Dimethylamine	2.71	0.005	0.96366	0.18696	NS
Trimethylamine	2.89	-0.005	0.96373	0.19130	NS
Creatine	3.02	0.002	0.98269	0.19565	NS
Pyruvate	2.35	-0.001	0.98871	0.20000	NS

* Characterization performed based on predicted hydrogen chemical shift using Chenomx software. FDR: False discovery rate; SIG: P < 0.05 and < FDR; NS: P > 0.05 and/or > FDR.

Table S6. Summary of metabolic pathways associated with aging after Metabolite Set Enrichment Analysis.

Metabolic Pathways	Total	Expected	Hits	P-value	Enrichment ratio*
Valine, leucine and isoleucine degradation	60	0.645	4	0.0026	6.20
Urea cycle	29	0.312	2	0.0364	6.41
Ammonia recycling	32	0.344	2	0.0437	5.81
Aspartate metabolism	35	0.376	2	0.0515	5.32
Lactose degradation	9	0.0967	1	0.0930	10.34
Malate-aspartate shuttle	10	0.107	1	0.1030	9.35
Arginine and proline metabolism	53	0.569	2	0.1070	3.51
Glucose-alanine cycle	13	0.14	1	0.1320	7.14
Spermidine and spermine biosynthesis	18	0.193	1	0.1780	5.18
Lactose synthesis	20	0.215	1	0.1960	4.65
Transfer of acetyl groups into mitochondria	22	0.236	1	0.2130	4.24
Glycolysis	25	0.269	1	0.2390	3.72
Beta-alanine metabolism	34	0.365	1	0.3120	2.74
Gluconeogenesis	35	0.376	1	0.3190	2.66
Galactose metabolism	38	0.408	1	0.3420	2.45
Sphingolipid metabolism	40	0.43	1	0.3560	2.33
Propanoate metabolism	42	0.451	1	0.3710	2.22
Steroidogenesis	43	0.462	1	0.3780	2.16
Steroid biosynthesis	48	0.516	1	0.4120	1.94
Glutamate metabolism	49	0.526	1	0.4180	1.90
Warburg effect	58	0.623	1	0.4750	1.61
Glycine and serine metabolism	59	0.634	1	0.4810	1.58
Tryptophan metabolism	60	0.645	1	0.4870	1.55
Bile acid biosynthesis	65	0.698	1	0.5160	1.43
Tyrosine metabolism	72	0.773	1	0.5530	1.29
Purine metabolism	74	0.795	1	0.5640	1.26

*Pathway enrichment ratio is computed by Hits/Expected Hits.
